# Dexterous Manipulation During Rhythmic Arm Movements in Mars, Moon, and Micro-Gravity

**DOI:** 10.3389/fphys.2018.00938

**Published:** 2018-07-17

**Authors:** Laurent Opsomer, Vincent Théate, Philippe Lefèvre, Jean-Louis Thonnard

**Affiliations:** ^1^System and Cognition Division, Institute of Neuroscience, Université catholique de Louvain, Louvain-la-Neuve, Belgium; ^2^Mathematical Engineering Department, Institute of Information and Communication Technologies, Electronics and Applied Mathematics, Université catholique de Louvain, Louvain-la-Neuve, Belgium; ^3^Cliniques Universitaires Saint-Luc, Physical and Rehabilitation Medicine Department, Université catholique de Louvain, Louvain-la-Neuve, Belgium

**Keywords:** precision grip, rhythmic movements, microgravity, Mars, Moon, object manipulation, motor adaptation

## Abstract

Predicting the consequences of one’s own movements can be challenging when confronted with completely novel environmental dynamics, such as microgravity in space. The absence of gravitational force disrupts internal models of the central nervous system (CNS) that have been tuned to the dynamics of a constant 1-*g* environment since birth. In the context of object manipulation, inadequate internal models produce prediction uncertainty evidenced by increases in the grip force (GF) safety margin that ensures a stable grip during unpredicted load perturbations. This margin decreases with practice in a novel environment. However, it is not clear how the CNS might react to a reduced, but non-zero, gravitational field, and if adaptation to reduced gravity might be beneficial for subsequent microgravity exposure. That is, we wondered if a transfer of learning can occur across various reduced-gravity environments. In this study, we investigated the kinematics and dynamics of vertical arm oscillations during parabolic flight maneuvers that simulate Mars gravity, Moon gravity, and microgravity, in that order. While the ratio of and the correlation between GF and load force (LF) evolved progressively with practice in Mars gravity, these parameters stabilized much quicker to subsequently presented Moon and microgravity conditions. These data suggest that prior short-term adaptation to one reduced-gravity field facilitates the CNS’s ability to update its internal model during exposure to other reduced gravity fields.

## Introduction

The human central nervous system (CNS) is highly skilled in its ability to model, with great accuracy, the physics underlying bodily interactions with the world. The construction of accurate internal models enables the brain to generate appropriate motor commands and to anticipate the consequences of the resulting movements, thereby allowing rapid actions to be performed despite the large delays inherent to the sensory feedback loop (Wolpert et al., 1995). In the context of dexterous object manipulation, such internal representations are of primary importance for fine tuning of grip force (GF) to the inertial and frictional properties of each object (Johansson and Westling, 1984, 1987a) and for feedforward modulation of that same GF to rapid fluctuations of the load force (LF) at the fingertips, including those induced by arm movements (Flanagan and Wing, 1993, 1995) or locomotion (Gysin et al., 2003). When exposed to novel dynamics, such as elastic or viscous force fields, internal models update rapidly to enable suitable adaptation of motor commands to the current context (Flanagan and Wing, 1997; Descoins et al., 2006).

On Earth, gravity is an omnipresent constraint with which our brains cope from birth. Thus, it is not surprising that our brains are able to anticipate its effects (Lacquaniti and Maioli, 1989; McIntyre et al., 2001; Papaxanthis et al., 2003, 2005) and use it as a reference frame (Papaxanthis et al., 1998b; Le Seac’h and McIntyre, 2007; Lopez et al., 2009; Clément et al., 2013) as well as a driving force (Papaxanthis et al., 1998a; White et al., 2008; Crevecoeur et al., 2009; Gaveau et al., 2016). During object manipulation, gravitational and inertial forces are both liable to induce slippage at the fingertips. In microgravity, LF decreases by an offset equal to the weight of the object. Consequently, the minimum GF required to avoid slippage is lower when the object is weightless than it would be on the ground, and the same anti-slippage safety margin can thus be achieved with less GF. However, when confronted with a 0-*g* environment for the first time (in the context of parabolic flights), subjects adopt the opposite strategy: they increase GF relative to that used in 1-*g* (Augurelle et al., 2003), thus producing an even greater safety margin. This initial increase in safety margin has been viewed as a strategy to cope with heightened uncertainty, or noise, in one’s ability to predict LF magnitudes (Crevecoeur et al., 2010; Hadjiosif and Smith, 2015). With training in the new gravitational field, the GF and safety margin decrease as subjects adapt to the novel gravitational field (Hermsdörfer et al., 1999; Augurelle et al., 2003).

It is not yet known whether experience with learning to adapt to one new gravity field can be transferred to other unknown gravity environments. We investigated this question by assessing upper-limb motor control in partial-gravity environments. Eight naïve subjects were exposed successively to Mars gravity, Moon gravity, and microgravity and we analyzed data collected while each performed rhythmic vertical arm movements with a handheld object while being exposed repeatedly to short periods (20–32 s) of each gravity level during parabolic flight maneuvers. It might be that the CNS builds a new internal model that is applied solely to each new gravity environment specifically. Alternatively, the CNS may be sufficiently flexible as to benefit from the development of a prior new internal model when adapting to the gravitational accelerations of subsequent new gravitational environments. If the former possibility is true, then the adaptation times of serial gravitational environments should be similar. If the latter is true, then the adaptation times should lessen in succession.

## Materials and Methods

### Subjects

Eight men (22–47 years old) participated in the present experiment. All subjects gave their informed consent and received approval to participate in parabolic flights from the National Center for Aerospace Medicine (class II medical examination). The experimental protocol was approved by the ESA Medical Board and by the local French Committee for Persons Protection. The experiment was carried out during the second Joint European Partial-*g* Parabolic Flights Campaign (JEPPFC) on board an Airbus A-300 ZERO-G aircraft. All subjects were naive to parabolic flight. They were given scopolamine to prevent motion sickness.

### Parabolic Maneuvers

Each flight consisted of a sequence of 31 parabolas. Each parabola began with ∼20 s of hyper-gravity (1.8 *g*) known as the pull-up phase, followed by a partial-gravity or microgravity phase of 32 s (Mars gravity), 25 s (Moon gravity), or 20 s (microgravity). The parabola ended with a second period of hyper-gravity known as the pull-out phase. The 31 parabolas were divided into three sessions of 13, 12, and 6 parabolas. The first session simulated Mars gravity (0.38 *g*), the second Moon gravity (0.16 *g*), and the last microgravity (0 *g*).

### Experimental Procedure

**FIGURE 1:**
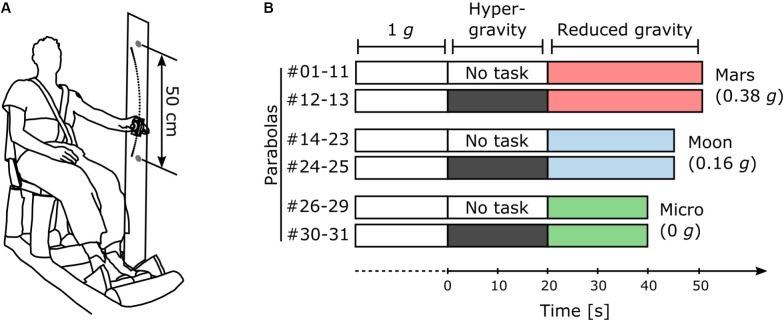
Protocol. **(A)** Illustration of the experimental setup. **(B)** Distribution of the 31 parabolas in the protocol among the three main sessions (*Mars*, *Moon*, and *Micro*). The subjects performed the task in hyper-gravity during the last two parabolas of each session.

Two subjects were tested simultaneously per flight. An opaque curtain separated the two subjects to prevent them from interacting. Each subject was seated in front of two visual targets (LEDs) fixed on a vertical bar 250 mm above and below shoulder level and at a distance of 800 mm from the chair back. Each subject was secured to his chair with straps (**Figure 1A**).

Each subject held a 260-*g* manipulandum between the thumb and index finger of his right hand with his right arm extended. They were asked to perform vertical oscillations of the extended arm at their preferred pace (i.e., a pace each subject felt to be spontaneous and comfortable).

Prior to flight, the subjects performed a training iteration of the experiment on the ground consisting of four blocks of at least 10 s each. On board the aircraft, they first performed four blocks of at least 10 s at 1 *g* during stationary flight, before the first parabola. Then, they performed the task during the 1-*g* period preceding each pull-up phase and during the reduced gravity phase of each parabola (**Figure 1B**). During the last two parabolas of each session, the subjects performed the task without interruption throughout the entire parabola, including the transition phases and the hyper-gravity phase (**Figure 1B**). The oscillations performed during the transition phases were not analyzed. After the last parabola, the subjects performed another four blocks of at least 10 s each during stationary flight.

### Data Collection

Gravitational acceleration was sampled at 800 Hz with a three-dimensional accelerometer (Analog Devices, ref. ADXL330). The three-dimensional (3D) position of the manipulandum was recorded at 200 Hz with a Codamotion tracking system (Charnwood Dynamics, Leicester, United Kingdom) and its acceleration was recorded at 800 Hz with a 3D accelerometer embedded in the manipulandum (Analog Devices, ref. ADXL330). Finally, 3D forces and torques were recorded at 800 Hz with Mini 40 force/torque transducers (ATI Industrial Automation, Apex, NC, United States) placed under each finger.

### Data Post-processing

Data post-processing was carried out with custom routines in MATLAB (Mathworks, United States). Position, acceleration, and force signals were filtered with a zero phase-lag Butterworth low-pass filter of order four with cutoff frequencies of 15 Hz, 10 Hz, and 15 Hz, respectively. Gravitational acceleration was filtered with the same type of filter, but with a cutoff frequency of 5 Hz.

**FIGURE 2:**
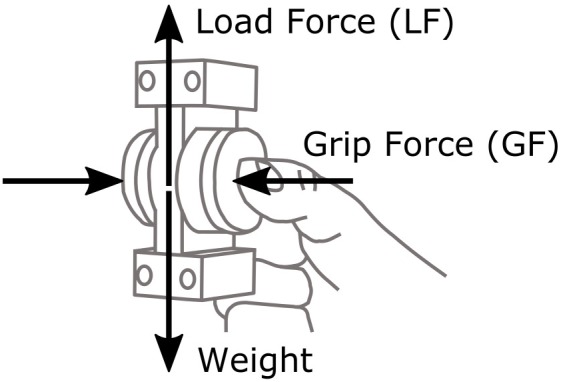
Illustration of the manipulandum, grip force (GF), and load force (LF).

Two forces were measured for the analysis of movement dynamics: GF exerted by the fingers normally to the contact surfaces, computed as the mean of the normal component of the forces recorded by the two force/torque sensors; and LF, that is, the vertical component of the tangential load relative to the manipulandum reference frame (**Figure 2**).

**FIGURE 3:**
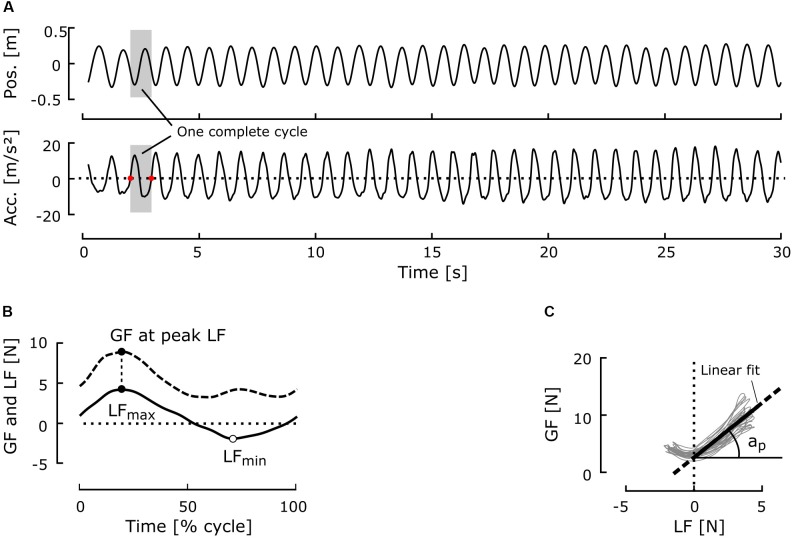
Typical time series and variables of interest. **(A)** Typical position and acceleration signals in one block. The gray area represents one complete cycle. The zeros of the acceleration signal during the downward movement of the arm (red dots) delimit the cycles. **(B)** Typical GF and LF traces during one cycle of oscillation. **(C)** GF plotted against LF for one complete block (33 cycles here). The thick line represents the linear regression computed for positive LF values with the ordinary least-squares procedure. The *R*^2^ coefficient and slope a_p_ were computed for each block from this linear regression.

One oscillation cycle was defined as the period between two consecutive minima of the vertical component of velocity relative to the aircraft reference frame (i.e., the first derivative of the vertical component of the position), which corresponds to the points at which the vertical component of the acceleration crosses zero during downward arm movement (**Figure 3A**). Cycles with amplitudes <20 cm were rejected (<1% of the cycles). When the mean gravitational acceleration of one cycle was out of pre-defined bounds (in *g*: *Micro*: [−0.1, 0.1]; *Moon*: [0.1, 0.3]; *Mars*: [0.3, 0.5]; *1-g*: [0.9, 1.2]), the corresponding cycle was also rejected (<5% of cycles per condition). Mean ± standard deviation (SD) cycle gravitational acceleration was 0.039 ± 0.02 *g* in microgravity, 0.19 ± 0.02 *g* in Moon gravity, 0.40 ± 0.03 *g* in Mars gravity, and 1.03 ± 0.04 *g* in Earth gravity.

Mean GF was computed for each cycle. To study how well GF was adjusted to LF, we computed the ratio between GF and LF for each cycle at the time of peak LF (**Figure 3B**). GF–LF correlation within the whole block was determined by calculating the coefficient of determination (*R*^2^) from linear regression modeling of the GF–LF relationship for positive values of LF (**Figure 3C**). The slope a_p_ of this regression was taken as the modulation gain of GF (**Figure 3C**).

### Statistical Analysis

To analyze the subjects’ adaptation to each gravitational condition, the effect of the repetition of blocks was tested independently for each condition with a linear mixed-effects model, which generalizes the concept of linear regression models (Brown and Prescott, 2006). Mixed-effects models allow between-subject variability to be accounted for in terms of model intercept and slope. The (numeric) variable *Block* was considered a fixed effect, while the factor *Subject* was considered a random effect that can affect the model’s intercept and slope. The variables studied were the frequency of arm oscillations, maximum and minimum LFs, the mean GF during each oscillation cycle, the GF/LF ratio at the time of peak LF, and the *R*^2^ coefficient of the linear regression between GF and LF for positive values of LF, computed for all cycles of one block.

Mathematically, the linear mixed-effects model with random intercept only (Model 1) can be written as

$$Y_{ij}=(b_{0}+\beta_{0j})+b_{1}\cdot X_{\text{i}}+\varepsilon_{\text{ij}},$$

where *Y_ij_* is here the value taken by the dependent variable on the *i*^th^ block of the *j*^th^ subject, *X*_i_ is the *i*^th^ block, β*_0j_* ∼*N*(0, σ_0_^2^) is the random intercept associated with the factor *Subject*, *b*_0_ and *b*_1_ are the fixed effects, and ε_i_*_j_* ∼*N*(0, σ^2^) are residuals. To account for between-subject variance in slope, a random slope associated with the factor *Subject*, β_1_*_j_* ∼*N*(0, σ_1_^2^), was introduced into the model, yielding Model 2:

$$Y_{\text{ij}}=(b_{0}+\beta_{0j})+(b_{1}+\beta_{1j})\cdot X_{\text{i}}+\varepsilon_{\text{ij}}.$$

Model 1 and Model 2 parameters were determined by maximum-likelihood estimation. Normal quantile–quantile plots were used to verify the normality of model residuals and random effects.

We used Model 2 for all variables and conditions. The effect of block repetition (parameter *b*_1_) was tested with Wald’s *t*-test. To assess whether the effect of block repetition differed significantly across subjects, Models 1 and 2 were compared based on Bayesian information criterion (BIC) values and with the likelihood ratio test (Brown and Prescott, 2006). To avoid overloading the text, only the likelihood ratio test results are reported here; they were always in agreement with the BIC values.

The next step was to evaluate the effect of gravity level on movement kinematics and dynamics. Blocks pertaining to the same gravity condition and to the same subject were pooled together, unless specified otherwise. We found no evidence that performing the task in hyper-gravity (parabolas nos. 12, 13, 24, 25, 30, and 31) affected task performance in the subsequent *Mars*, *Moon*, or *Micro* conditions [two-way repeated-measures analysis of variance (ANOVA) with the factors *Condition* and *Preceded by Hyper*], with the exception of movement amplitude, which was significantly smaller during these parabolas [*F*_(1,7)_ = 5.64, *p* = 0.049]. Because this effect was small (η^2^ = 0.065, difference in means of 2.6 mm) and did not affect peak LF, these parabolas were included in this analysis. The effect of gravity level was investigated with a one-way repeated-measures ANOVA. Generalized eta-squared is reported for effect size. Huynh–Feldt correction was used when the condition of sphericity was violated (Mauchly’s test for sphericity, 0.05 level). When the omnibus test revealed a significant effect of gravity condition, Tukey’s pairwise multiple comparison test was used.

## Results

### Typical Traces

**FIGURE 4:**
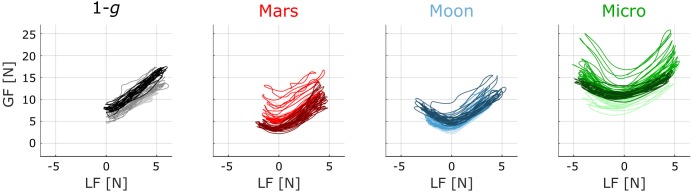
Traces of GF versus LF for a typical subject within each condition. Lighter traces correspond to the first block, medium traces to the third block, and darker traces to the last block of the condition. Only the 15 first cycles are plotted. In the *1-g* condition, the blocks plotted are the first, third, and fourth blocks performed on board the aircraft before the first parabola.

Typical traces of GF plotted against LF for one subject are presented in **Figure 4**. The 15 first cycles of the first (lightest color), third (normal color), and last (darkest color) blocks within the *1-g*, *Mars*, *Moon*, and *Micro* conditions are plotted. These traces provide qualitative illustrations of the main results of this study. GF and LF show a clear correlation in each condition, from the very first block, even in microgravity. In 1-*g*, LF was mainly positive and the relationship between LF and GF was essentially linear. As gravity decreased in the subsequent *Mars*, *Moon*, and *Micro* conditions, the LF values shifted toward negative values while the movement kinematics were unchanged. For negative values of LF, the correlation between the two forces became negative. Indeed, to avoid slippage of the object, GF must increase when the absolute value of the load acting on the fingertips increases, whatever the direction of the load.

There was more variance in the GF profile during early exposure to Mars gravity (light red traces) than during the last block of exposure to Mars gravity (darkest red trace). By contrast, the first block of the *Moon* condition presents more reproducibility in GF modulation, suggesting that the subject adapted much quicker to this second novel gravitational field. Lastly, GF appeared to be higher in microgravity than in the *Mars* and *Moon* conditions, suggesting that the subject felt the need to apply a stronger grip to avoid dropping the object, although the maximum LF did not increase in microgravity.

### Adaptation to Different Gravitational Levels

The (vertical) amplitude of arm movement (imposed by target LEDs 50 cm apart) was 56.7±6.4 cm (Mean ± SD) across all subjects, conditions, and blocks. Movement amplitude was not affected by gravity condition [*F*_(4,28)_ = 2.0, *p* = 0.12, η^2^ = 0.057] and did not evolve significantly across blocks, as revealed by the linear mixed-effects models (*Mars*: *b*_1_ = 1.5 × 10^−3^, *t*_79_ = 1.08, *p* = 0.28; *Moon*: *b*_1_ = 6.0 × 10^−5^, *t*72 = 0.05, *p* = 0.96; *Micro*: *b*_1_ = 1.6 × 10^−3^, *t*_21_ = 0.27, *p* = 0.79).

**FIGURE 5:**
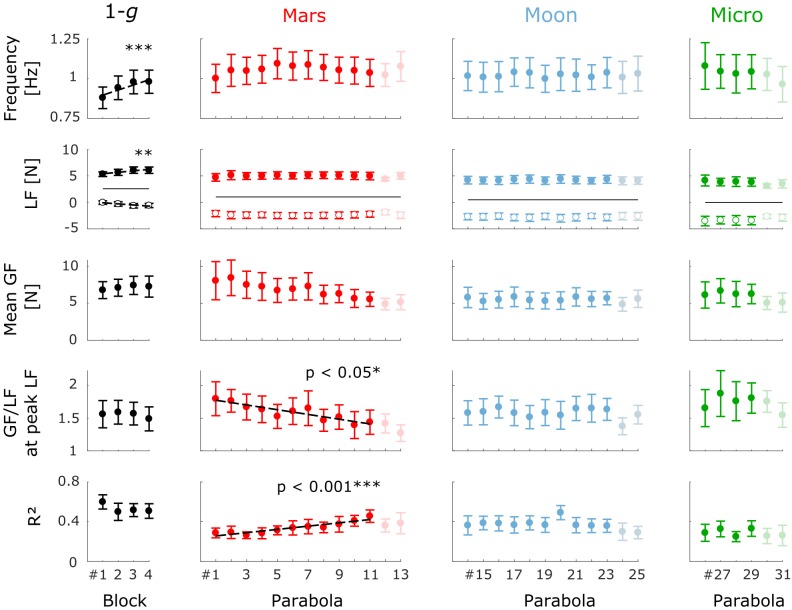
Mean (±standard error [*N* = 8]) kinematic and dynamic variable values by block number within each gravity condition. From top to bottom: movement frequency, LF minimum (open circles), and LF maximum (filled circles), mean GF, GF/LF ratio at the time of peak LF, and *R*^2^ coefficient. The solid black lines in the LF plots represent the weight of the manipulandum. Blocks plotted in the *1-g* condition are the four first blocks performed on board the aircraft. The regression lines estimated by the mixed-effects models are plotted (dashed lines) where the effect of block number is significant (^∗^*p* < 0.05; ^∗∗^*p* < 0.01; ^∗∗∗^*p* < 0.001). The last two parabolas of each session (faint dots) were not included in the analysis (see the section “Materials and Methods”).

Plots of the study cohorts’ mean kinematic and dynamic variable values for each block within each condition are shown in **Figure 5**. Regression lines that estimate the fixed effect are plotted when the slope is significantly different from zero. Each gravity condition is detailed hereafter.

#### Earth Gravity (1 *g*)

Frequency increased significantly across the four preliminary stationary-flight blocks (*b*_1_ = 0.033, *t*_23_ = 4.74, *p* < 0.001; **Figure 5**), but neither the GF/LF ratio at the time of peak LF nor the *R*^2^ coefficient evolved significantly across these blocks (*b*_1_ = −0.024, *t*_23_ = −0.88, *p* = 0.39 and *b*_1_ = −0.025, *t*_23_ = −1.20, *p* = 0.24, respectively). We can therefore assume that the variables characterizing the arm-hand coordination were stable at 1 *g* before the task was performed in the *Mars* condition. Subjects also performed the task during the 1-*g* period preceding each parabola (not shown). Again, the variables were essentially stable across these 31 blocks (*p* > 0.5 for mean GF, GF/LF ratio at peak LF and *R*^2^). Again, no effect of block number was detected after the last parabola when subjects performed the task during stationary flight for the four final blocks (not shown). No differences were found between those last four blocks and the four first blocks performed in 1 *g* in terms of mean GF, GF/LF ratio and *R*^2^ coefficient (paired *t*-test; *p* > 0.4), confirming that motor adaptation was complete after the four first blocks performed during stationary flight.

#### Mars Gravity (0.38 *g*)

Because the subjects performed the oscillations at their own pace, the number of cycles within each block varied across subjects. In the *Mars* condition, the subjects performed 32.5±8.2 oscillation cycles per block (Mean ± SD).

The first block performed under Mars gravity was the first experience of reduced gravity for all subjects. As expected, we observed a significant effect of block repetition on two dependent variables that characterize the dynamics of precision grip as the subjects adapted to this new experience. The GF/LF ratio at the time of LF maximum decreased significantly from block 1 to block 11 (*b*_1_ = −0.036, *t*_79_ = −2, *p* < 0.05), as depicted in **Figure 5**, and there was a significant variance in slope across subjects (*SD* = 0.046, $\chi_{2}^{2}$ = 17.4, *p* < 0.001). Estimated slopes and intercepts of the linear model for each subject are reported in **Table 1**. Six of the eight subjects presented an overall decrease in GF/LF ratio. The linear mixed-effects model estimates that the subjects decreased their GF/LF ratio by 20%, on average, across the eleven blocks of the *Mars* condition. The decrease in the GF/LF ratio at the moment of maximum load (when risk of object slippage is greatest) indicates that motor control of the precision grip improved with time by progressively minimizing excess GF beyond that needed to ensure a stable grip. Note that the slope of the decrease in mean GF was not significantly different from zero (*b*_1_ = −0.265, *t*_79_ = −1.76, *p* = 0.08) and varied significantly across subjects (*SD* = 0.41, $\chi_{2}^{2}$ = 64.7, *p* < 0.001).

**Table 1:** Estimates of slopes and intercepts for a block repetition effect on GF/LF ratio for each subject in the *Mars* condition.

Subject	Slope (*b*_1_) [Block^−1^]	Intercept (*b*_0_) [−]
1	−0.0076	2.25
2	0.0074	0.86
3	−0.021	2.37
4	−0.125	2.66
5	−0.040	1.94
6	−0.069	1.50
7	0.0072	1.41
8	−0.041	1.46

In parallel, the correlation between GF and LF (*R*^2^) increased significantly with block repetition (*b*_1_ = 0.016, *t*_79_ = 3.76, *p* < 0.001; see **Figure 5**). The slope of the linear model did not vary significantly across subjects (*SD* = 7.5 × 10^−3^, $\chi_{2}^{2}$ = 4.49, *p* = 0.11). From the first to the last block, *R*^2^ increased by an average of 66%, as estimated by the linear mixed-effects model.

Because the coefficient *R*^2^ characterizes the coordination between arm kinematics and the dynamics of prehension, the presently observed increase in *R*^2^ may reflect an actual increase in force correlation within each cycle owing to improved arm-hand coordination. Decreased intra-block variability of the mean GF and/or GF modulation gain may also account for or contribute to the increased *R*^2^. Additional analyses showed that none of these three possible effects were significant, suggesting that the increase in global intra-block *R*^2^ was probably due to a combination of these three effects given that the number of oscillations performed (and selected) per block did not vary across blocks (*b*_1_ = −0.035, *t*_79_ = −0.14, *p* = 0.89). Overall, these results reflect an increase in movement reproducibility as subjects adapted to a new gravitational field.

In contrast, the frequency (**Figure 5**) and GF modulation gain, a_p_ (not shown), did not evolve across blocks (*b*_1_ = 1.8 × 10^−3^, *t*_79_ = 0.30, *p* = 0.76 and *b*_1_ = 3.8 × 10^−3^, *t*_79_ = 0.39, *p* = 0.69; respectively). Again, the slope of the model varied significantly across subjects (frequency: *SD* = 0.016, $\chi_{2}^{2}$ = 57.0, *p* < 0.001; a_p_: *SD* = 0.026, $\chi_{2}^{2}$ = 32.0, *p* < 0.001).

#### Moon Gravity (0.16 *g*)

In the *Moon* condition (parabolas 14–23), the subjects performed 24.2 ± 7.0 cycles per block (Mean ± SD). Unlike the preceding *Mars* condition, there was no significant effect of block repetition on GF/LF ratio at the time of peak LF (*b*_1_ = 4.6 × 10^−5^, *t*_72_ < 0.01, *p* > 0.99). This negative finding was consistent across all subjects (*SD* = 0.021, $\chi_{2}^{2}$ = 2.88, *p* = 0.24). Moreover, the *R*^2^ coefficient did not change significantly across blocks (*b*_1_ = −2.0 × 10^−3^, *t*_72_ = −0.53, *p* = 0.60; *SD* = 5 × 10^−3^, $\chi_{2}^{2}$ = 0.95, *p* = 0.62) and we did not observe any significant effects of block repetition on any of the examined variables. This stability across blocks suggests that the subjects adapted to the new gravitational field within the duration of one block (<25 s).

#### Microgravity (0 *g*)

In microgravity, the subjects performed 20.1 ± 6.5 cycles per block (Mean ± SD). Even though all subjects were experiencing microgravity for the very first time, there were no significant effects of block number (parabolas 26–29) on mean GF (*b*_1_ = −0.063, *t*_21_ = −0.33, *p* = 0.75), LF/GF ratio at the time of peak LF (*b*_1_ = 0.020, *t*_21_ = 0.46, *p* = 0.65), or *R*^2^ (*b*_1_ = 3.5 × 10^−3^, *t*_21_ = 0.19, *p* = 0.85). However, the GF/LF ratio increased in microgravity, as will be emphasized below, a finding inconsistent with the conclusion that adaptation was complete after less than one block of training. Nevertheless, performance was close to that in the *Moon* condition.

### GF Adapts Adequately to Gravity Changes

**FIGURE 6:**
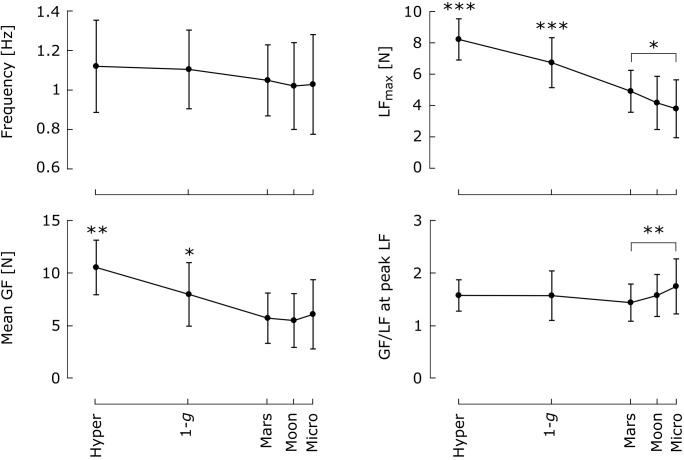
Mean and 95% confidence interval of the mean (*N* = 8) of frequency, maximum LF, mean GF and GF/LF ratio at the time of peak LF as a function of gravity condition. Means that differ significantly across conditions are marked with an *asterisk* (^∗^*p* < 0.05; ^∗∗^*p* < 0.01; ^∗∗∗^*p* < 0.001). Blocks within the same condition and subject were pooled. Only the last six blocks were selected in the *Mars* condition, and the first two blocks performed in 1 *g* on board the aircraft were excluded from the analysis of movement frequency.

**Figure 6** presents the effects of gravity level on frequency, mean GF, and GF/LF ratio at peak LF. Blocks from the same subject and condition were pooled, except in the *Mars* condition, where only the last six blocks (parabolas 8–13) were selected to avoid learning-phase artifacts. For the same reason, the first two blocks performed in 1 *g* on board the aircraft were withdrawn from the movement frequency analysis. Note that the last two parabolas of each session (where subjects performed the task during the entire duration of the parabola) were included in this analysis. No effect of block repetition was found in the hyper-gravity condition; those blocks were therefore pooled as well.

Gravity level did not affect oscillation frequency [*F*_(4,28)_ = 2.29, *p* = 0.84, η^2^ = 0.027]. In addition to altering peak LF [*F*_(4,28)_ = 57.5, *p* < 0.001, η^2^ = 0.47], gravity also had a strong effect on mean GF [*F*_(4,28)_ = 20.5, *p* < 0.001, η^2^ = 0.27] and a relatively moderate effect on GF/LF ratio at the time of peak LF [*F*_(3.21,22.5)_ = 3.1, *p* < 0.044, η^2^ = 0.04]. Mean GF in 1 *g* was significantly higher than that in Mars gravity, Moon gravity, and microgravity (Tukey’s *post hoc*: *p* < 0.05 in all three cases) but lower than that in hyper-gravity (*p* = 0.001). Hence, mean GF was also significantly higher in hyper-gravity than in partial- and microgravity (*p* < 0.001 for the three comparisons). No differences were found in GF/LF ratio at the time of peak LF between any of the *Hyper*, *1-g*, *Mars*, and *Moon* conditions, suggesting that grip control was equivalently adapted in all four conditions, after the adaptation process that occurred in Mars gravity. However, the GF/LF ratio was significantly more elevated in microgravity than in Mars gravity (*p* = 0.004).

## Discussion

In this study, we found that prior (short-term) adaptation to Mars gravity quickened subsequent adaptation to Moon and 0-*g* environments, suggesting that the new internal model developed during exposure to Mars gravity is flexible enough to be adapted rapidly to other novel reduced gravitational fields. The rapid adaptation of the control of prehension dynamics in subsequent environments after practice is consistent with a transfer of learning from the *Mars* condition to the subsequent *Moon* and *Micro* conditions. Importantly however, in microgravity the subjects applied a higher GF/LF ratio than in the *Mars* condition, producing a greater safety margin against slippage, which suggests that the adaptation to weightlessness may have been incomplete.

Although all eight subjects were experiencing reduced gravity for the very first time, the temporal coupling between GF and LF and the GF modulation gain adapted rapidly (in <1 parabola) to the novel environmental dynamics of the reduced gravity conditions (0.38 *g*, 0.16 *g*, and 0 *g*). These observations are in line with previous studies showing preserved synchronization between GF and LF during arm oscillations in weightlessness (Hermsdörfer et al., 2000; Augurelle et al., 2003) and reinforce the view that control of average GF and GF modulation are independent (Flanagan and Wing, 1993; Augurelle, 2002; Nowak et al., 2002; Nowak and Hermsdörfer, 2003).

During the first parabola simulating Mars gravity, the subjects tended to apply an excessive GF relative to the peak LF. They then learned throughout the 11-block session that they could safely loosen their grip. Similar, albeit more pronounced, practice-associated decreases in GF were observed previously in 0-*g* stationary holding (Hermsdörfer et al., 1999) as well as during rhythmic (Augurelle et al., 2003) and discrete (Crevecoeur et al., 2009, 2010) arm movements performed while holding an object. The influence of the stress and excitement induced by an uncommon context such as parabolic flight should not be ignored given that stress-related hormones have been shown to increase in subjects undergoing parabolic flight maneuvers (although only in subjects that experienced motion sickness; Schneider et al., 2007). Notwithstanding, the increases in GF observed under reduced gravity conditions likely constitute a strategy to establish a higher safety margin to compensate for the uncertainty induced by an inaccurate internal model of the physics of the environment (Crevecoeur et al., 2010). Indeed, GF control during reaching movements perturbed by a viscous force field of variable intensity was shown to be more sensitive to LF variability than to mean LF (Hadjiosif and Smith, 2015), supporting the idea that uncertainty *per se* favors augmentation of the safety margin against slippage. As subjects integrate the dynamics of the novel environment into a new internal model, the uncertainty in load prediction should decrease, reflected by a decreasing GF/LF ratio with further task repetition. Moreover, we observed a highly significant increase in GF–LF correlation (decrease in the intra-block variance of the GF profile) across the eleven blocks of the *Mars* condition as subjects learned the dynamics of the new gravitational field, consistent with a progressive reduction in LF-prediction noise.

In contrast, and most interestingly, during the subsequent *Moon* condition, mean GF, GF/LF ratio at the time of peak LF, and GF/LF correlation were stabilized from the first parabola onward. Moreover, these variables were stable across the four microgravity blocks that followed the *Moon* condition. This is coherent with our hypothesis that there has been a transfer of learning from one environment to the next. Although the increase in GF/LF ratio that we observed in microgravity, relative to that in Mars gravity, suggests that adaptation to microgravity might have been incomplete, it should be emphasized that the effect was quite small (+21% vs. Mars gravity, +11% vs. 1 *g*) relative to the results of Augurelle et al. (2003), in which the safety margin (GF/LF ratio at peak LF minus half of the inverse of the coefficient of friction) was more than double that in 1 *g* during the first parabola in microgravity. Their protocol was similar to ours except that subjects did not perform the task in partial gravity beforehand, suggesting that performing the task in a prior partial gravity attenuates this reactive increase in safety margin exhibited in weightlessness.

This contrast with the results of Augurelle et al. (2003) is a strong indication that training in partial gravity might actually be sufficient to reach the level of adaptation typically observed after 5–10 parabolas (Hermsdörfer et al., 1999; Augurelle et al., 2003; Papaxanthis et al., 2005; Crevecoeur et al., 2009, 2010). This apparent transfer effect is not trivial given previous suggestions that when performing rhythmic arm movements in microgravity, the CNS may rely on a different motor strategy than when performing the same movements under the Earth’s gravity, hyper-gravity, or partial gravity (White et al., 2008). More precisely, the presence of gravity could allow the CNS to rely on central pattern generators to produce rhythmic movements that are tuned to the resonant frequency of the arm-object system. And indeed, White et al. (2008) showed that the frequency of rhythmic arm movements is close to the estimated resonant frequency of the system. In weightlessness, however, central pattern generators can no longer rely on gravity to initiate movements and a new, higher-level strategy must be established. This change translates into a disruption of the frequency-gravity relationship in 0 *g*. Our results show that the implementation of this new strategy for the control of arm kinematics in microgravity does not necessarily alter the internal models used for the control of prehension dynamics, which were updated previously in the Mars and Moon gravity conditions.

Despite the apparent immediate adaptation of the subjects to weightlessness, it appears that GF control may not be as finely tuned in microgravity as in Mars gravity. Indeed, GF/LF ratio at the time of maximum LF was moderately but significantly higher in the *Micro* condition than in the *Mars* condition. Generally, GF scaled to peak LF across gravity conditions, except in microgravity where mean GF was not decreased relative to the *Mars* or *Moon* conditions, leading to a slightly elevated GF/LF ratio. This divergence could mean that the adaptation to microgravity was incomplete, and that a second learning phase would be observed with longer exposure. In altered gravity, subjects tend to generate excessive isometric forces when instructed to produce forces of specific amplitude and direction (Bock and Cheung, 1998; Girgenrath et al., 2005; Mierau et al., 2008). Mierau et al. (2008) found no evidence that degraded segmental excitability or proprioception could explain this impoverishment in force estimation, and proposed that the cause should be looked for at a higher level. As mentioned above, Crevecoeur et al. (2010) suggested that altered movement kinematics and dynamics in 0 *g* could be explained by elevated prediction noise. That is, our CNSs may cope with greater LF uncertainty by increasing the GF safety margin (Hadjiosif and Smith, 2015). Further experiments under long-term exposure to microgravity in space should be carried out to investigate whether prediction uncertainty can be reduced to levels observed in the Earth’s gravitational field. Notwithstanding, it is important to recognize that the increased safety margin in the first 0-*g* parabola was small (20%) relative to the results of Augurelle et al. (2003) (>100%), and the difference was only significant compared to the Mars gravity condition.

This study had some limitations. First, during the last two parabolas of the *Mars*, *Moon*, and *Micro* sessions, the subjects performed the task during the hyper-gravity phase preceding the reduced gravity phase. We cannot exclude the possibility that these trials impacted subsequent performance. However, intuitively, it makes more sense to attribute the quick motor-control adaptation in Moon gravity to the prior adaptation in Mars gravity than to practice in hyper-gravity. Second, due to restrictions inherent to parabolic flight protocols, only four microgravity blocks (after discarding the fifth and sixth blocks) were available for the analysis of motor adaptation across blocks. As a result, the statistical power of testing the effect of block repetition was reduced compared to that in the *Mars* and *Moon* conditions. Nevertheless, the constancy of the mean GF across the four blocks is striking given that previous studies showed a substantial decrease in GF over the first 5–10 blocks performed in weightlessness (Hermsdörfer et al., 1999; Augurelle et al., 2003; Crevecoeur et al., 2009). Third, we did not monitor subjects’ stress during the experiment, given the complex and invasive aspect of the measurement procedure. Schneider et al. (2007) observed an increase of stress-related hormones during the course of a parabolic flight but only in subjects experiencing motion-sickness. During our experiment, two out of eight participants experienced motion-sickness, which lowers the potential impact of stress on the results. Finally, we did not account for possible variations in the coefficient of finger pad-object contact friction. The minimum GF required to avoid slippage is inversely proportional to the static coefficient of friction; accordingly, a change in friction leads GF adjustment to maintain a constant safety margin (Johansson and Westling, 1987b). In addition, the static coefficient of friction is influenced by the applied normal force and skin moisture (Adams et al., 2007, 2013; Andre et al., 2011; van Kuilenburg et al., 2013; Barrea et al., 2016). Thus, it might be argued that the effect of block repetition or gravity on the GF/LF ratio may be a hidden effect of friction variation. In the present experiment though, GF at the time of peak LF exceeded 5 N for 80% of the cycles. Generally, at that level, the influences of normal force and moisture on friction become negligible (André et al., 2009; Barrea et al., 2016). But even if the influence of friction variations cannot be entirely neglected, friction alone cannot explain the significant increase in GF–LF correlation observed in the *Mars* condition, nor the performance constancy observed in the *Moon* and *Micro* conditions.

## Conclusion

Our results show that experiencing partial gravity before microgravity may be sufficient to bypass the high increase in uncertainty typical of early exposure to weightlessness evidenced by the use of excessive GF. Prior exposure to partial gravity may produce a more accurate and flexible internal model, in addition to reducing stress and load-prediction noise, though that noise may remain slightly higher than in 1 *g*. While these results only apply to short, repeated exposures, we can speculate that if short-term exposure to Mars gravity facilitates motor adaptation to Moon gravity, long-term exposure likely does as well. One way to progress into the understanding of learning transfer across gravity levels could be to inverse the order of gravity changes: starting with 0 *g*, then 0.16 *g* and finally 0.38 *g*. This could help us in studying whether microgravity is indeed a “singularity” from the point of view of motor control. In addition, the current study was restrained to rhythmic movements: it would be relevant to perform a similar study with discrete movements, which are thought to rely on a higher-level control (Schaal et al., 2004). The present evidence of motor learning transfer from one partial gravity environment to another may be useful for the development of future training programs for astronauts.

## Author Contributions

J-LT, PL, and VT contributed to the conception and design of the study. LO performed data post-processing and statistical analysis and wrote the manuscript. All authors contributed to manuscript revision, and read and approved the submitted version.

## Conflict of Interest Statement

The authors declare that the research was conducted in the absence of any commercial or financial relationships that could be construed as a potential conflict of interest.
